# Supplementary figures and images for: The transcription factor HLH-26 controls probiotic-mediated protection against intestinal infection through up-regulation of the Wnt/BAR-1 pathway
Source: PLoS Biol. 2022 Mar 9;20(3):e3001581. doi: 10.1371/journal.pbio.3001581 (PMC8936500; doi:10.1371/journal.pbio.3001581)

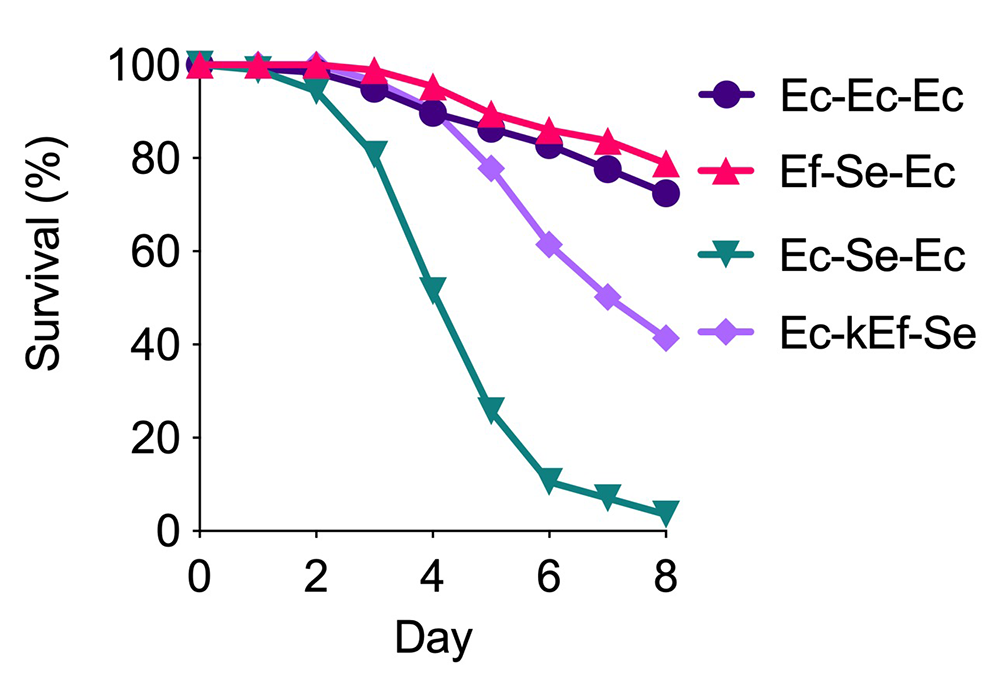

Supplement: S1 Fig — Survival curve from a treatment–1-day infection assay showing that both live and heat-killed E. faecium inhibits S. enterica pathogenesis. Animals were treated on live or heat-killed E. faecium for 1 day before infection with S. enterica for 1 day. Animals were then transferred onto E. coli OP50 plates for the rest of the assay, and survival was scored. The data underlying all the graphs shown in the figure can be found in S1 Data. (TIF) [file pbio.3001581.s001.tif]

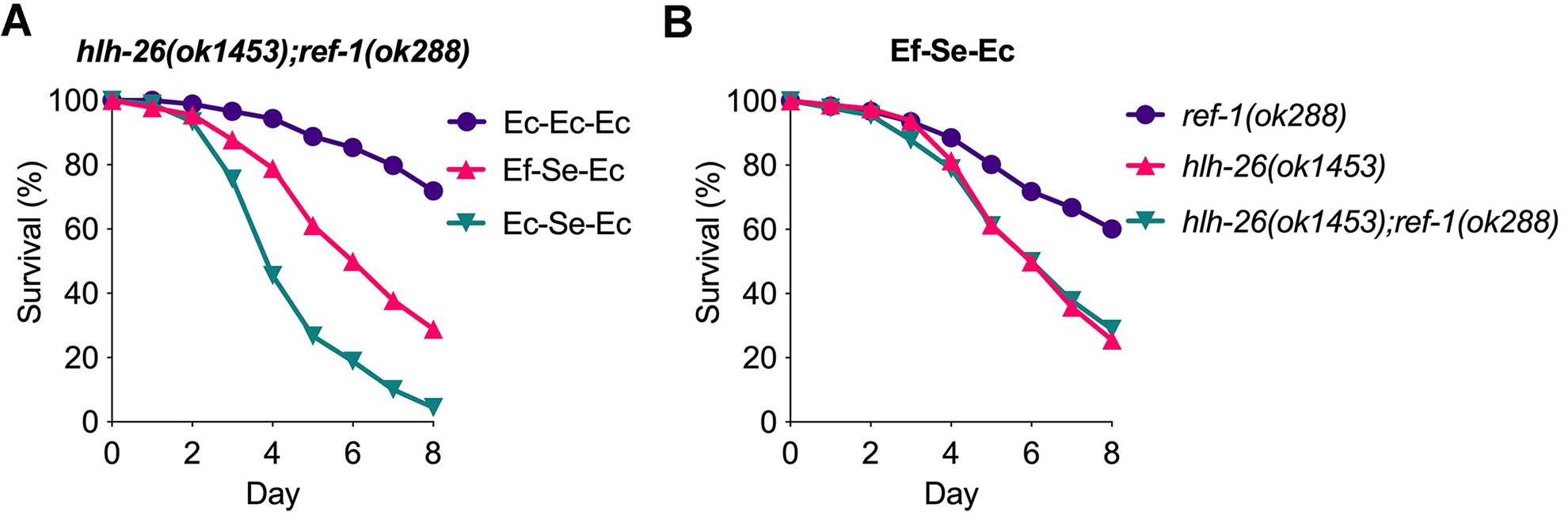

Supplement: S2 Fig — (A) Survival curve assaying E. faecium–mediated protection of hlh-26(ok1453);ref-1(ok288) double mutant. Survival curves are representative assays of 3 independent experiments. n = 60 to 90. (B) Survival curves of ref-1(ok288), hlh-26(ok1453), and hlh-26(ok1453);ref-1(ok288) animals grown on E. faecium for 1 day, infected with S. enterica for 1 day, and transferred to E. coli. ref-1(ok288) animals versus hlh-26(ok1453), P < 0.0001; ref-1(ok288) animals versus hlh-26(ok1453);ref-1(ok288) animals, P < 0.0001. hlh-26(ok1453) animals versus hlh-26(ok1453);ref-1(ok288) animals, P = NS. The data underlying all the graphs shown in the figure can be found in S1 Data. Ec, E. coli OP50; Ef, E. faecium; NS, nonsignificant; Se, S. enterica. (TIF) [file pbio.3001581.s002.tif]

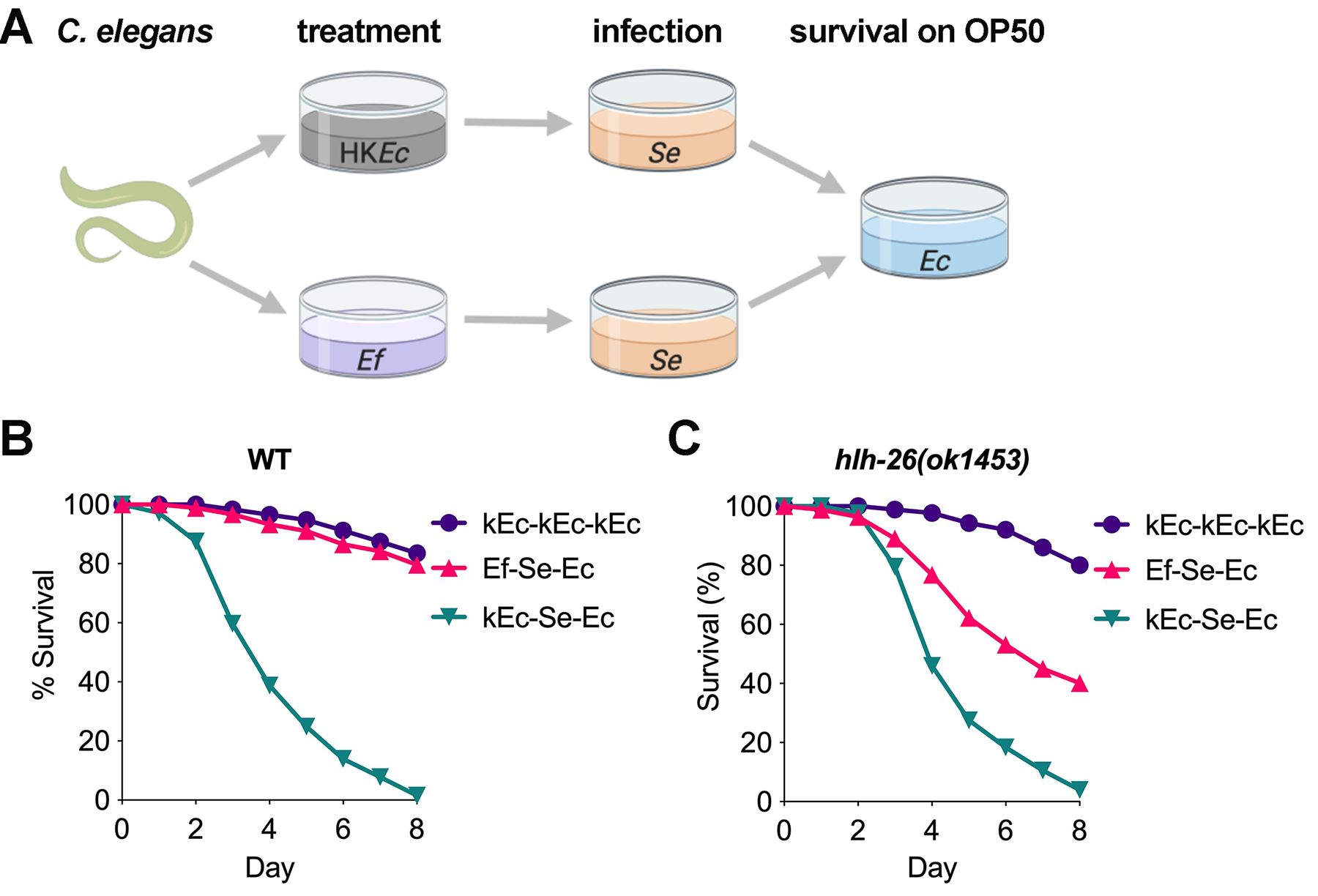

Supplement: S3 Fig — (A) Schematic of the E. faecium treatment and 1-day infection assay. Animals were grown on E. faecium lawns for 1 day before infection with S. enterica for 1 day. Animals were then transferred onto E. coli OP50 plates for the remainder of the assay, and survival was scored. Control animals were fed heat-killed E. coli OP50 throughout the assay. Survival curves assaying E. faecium–mediated protection in (B) wild-type and (C) hlh-26(ok1453) animals. The data underlying all the graphs shown in the figure can be found in S1 Data. Ec, E. coli OP50; Ef, E. faecium; Se, S. enterica; WT, wild-type. (TIF) [file pbio.3001581.s003.tif]

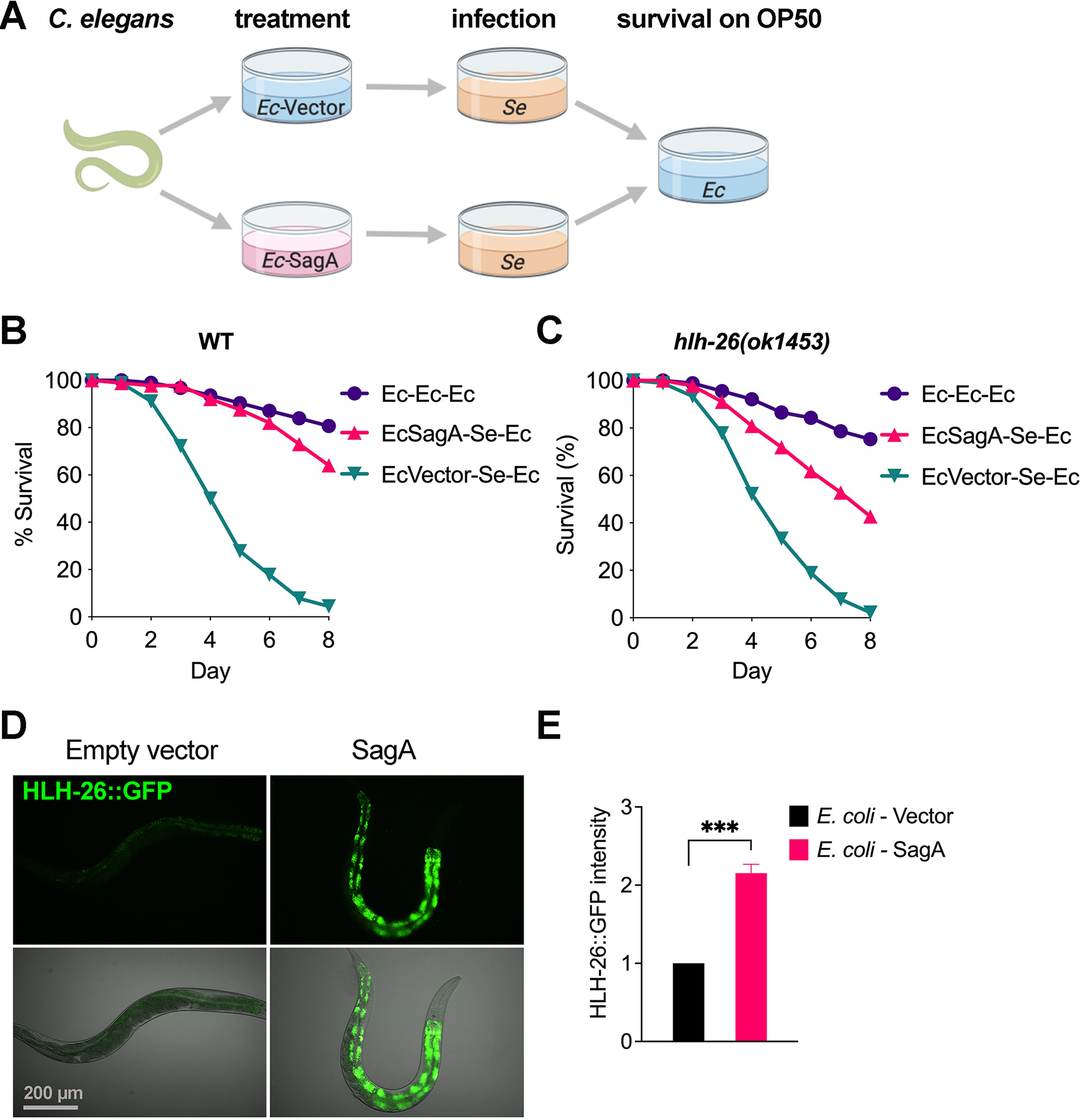

Supplement: S4 Fig — (A) Schematic of the E. faecium treatment and 1-day infection assay. Animals were pretreated with E. coli BL21 harboring empty vector or expressing SagA followed by S. enterica infection. Animals were then transferred onto E. coli OP50 plates for the remainder of the assay, and survival was scored. Survival curves assaying SagA-mediated protection in (B) wild-type and (C) hlh-26(ok1453) animals. (D) Fluorescence micrographs of animals expressing GFP fused to HLH-26 (HLH-26::GFP) after 24-h fed on E. coli BL21 harboring empty vector or expressing SagA. (E) Quantification of the animals shown in (D), N = 3 biological replicates. ***P < 0.001. The data underlying all the graphs shown in the figure can be found in S1 Data. Ec, E. coli OP50; EcSagA, E. coli BL21 expressing SagA; EcVector, E. coli BL21 harboring empty vector; Se, S. enterica. (TIF) [file pbio.3001581.s004.tif]

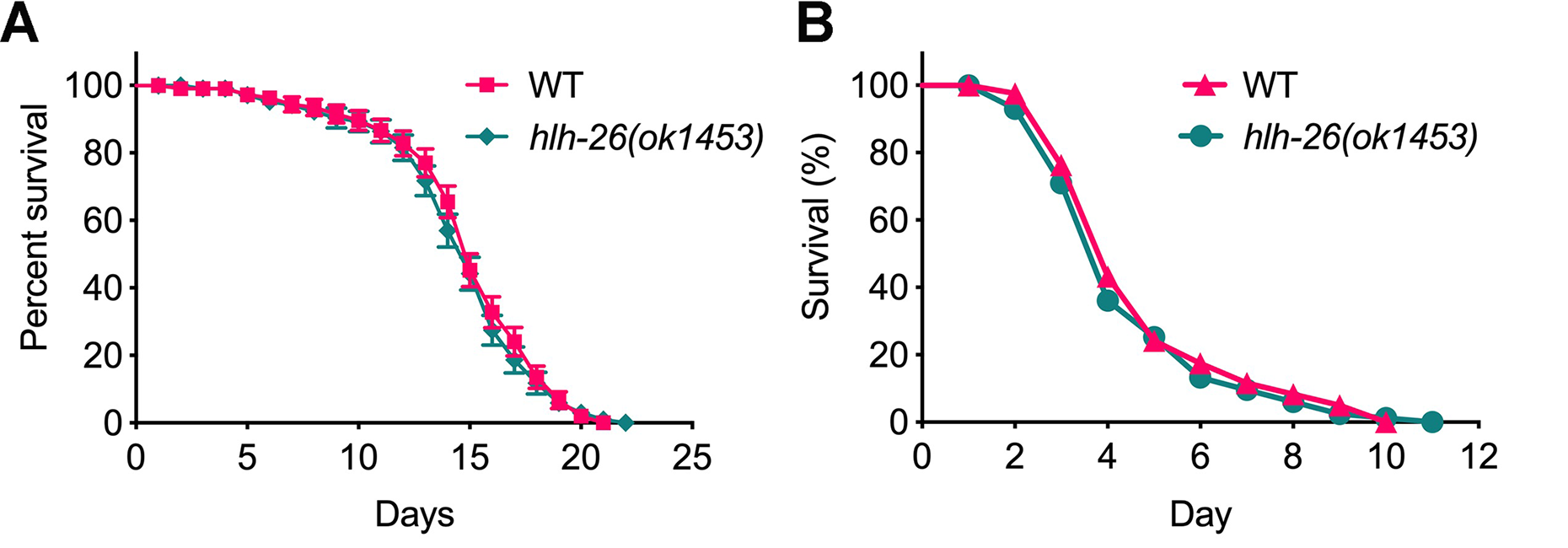

Supplement: S5 Fig — (A) WT and hlh-26(ok1453) animals were grown on E. faecium and scored for survival. WT animals versus hlh-26(ok1453), P = NS. (B) WT and hlh-26(ok1453) animals were grown on S. enterica and scored for survival. WT animals versus hlh-26(ok1453), P = NS. The data underlying all the graphs shown in the figure can be found in S1 Data. NS, nonsignificant; WT, wild-type. (TIF) [file pbio.3001581.s005.tif]

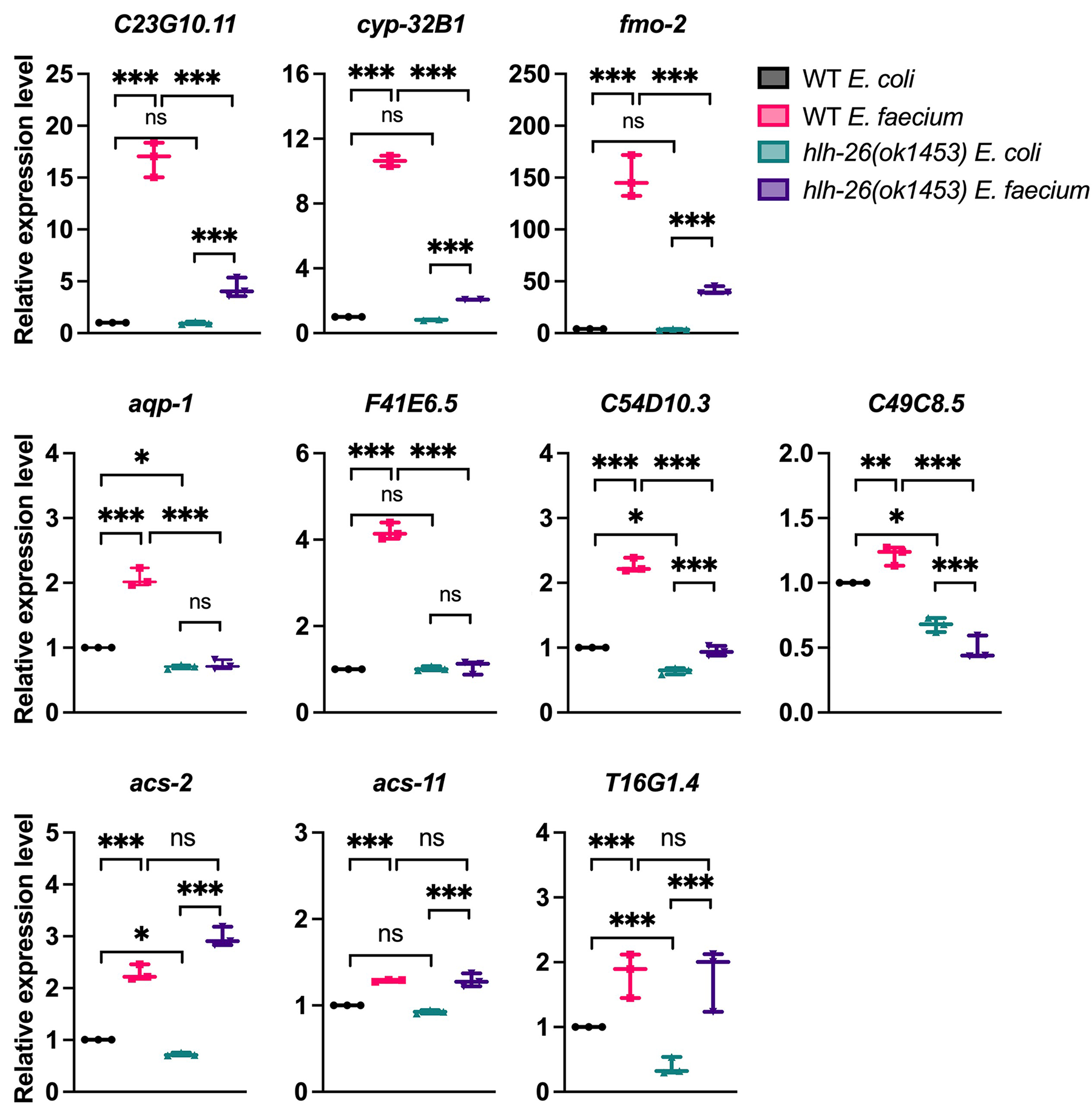

Supplement: S6 Fig — qRT-PCR validation of selected coexpressed genes from which the upstream regulator hlh-26 was predicted. Values are expressed as the fold difference compared with wild-type worms fed on heat-killed E. coli ± SD by one-way ANOVA with Tukey’s multiple comparisons test, *P < 0.05, **P < 0.01, ***P < 0.001. The data underlying all the graphs shown in the figure can be found in S1 Data. ns, nonsignificant; qRT-PCR, quantitative reverse transcription PCR; WT, wild-type. (TIF) [file pbio.3001581.s006.tif]

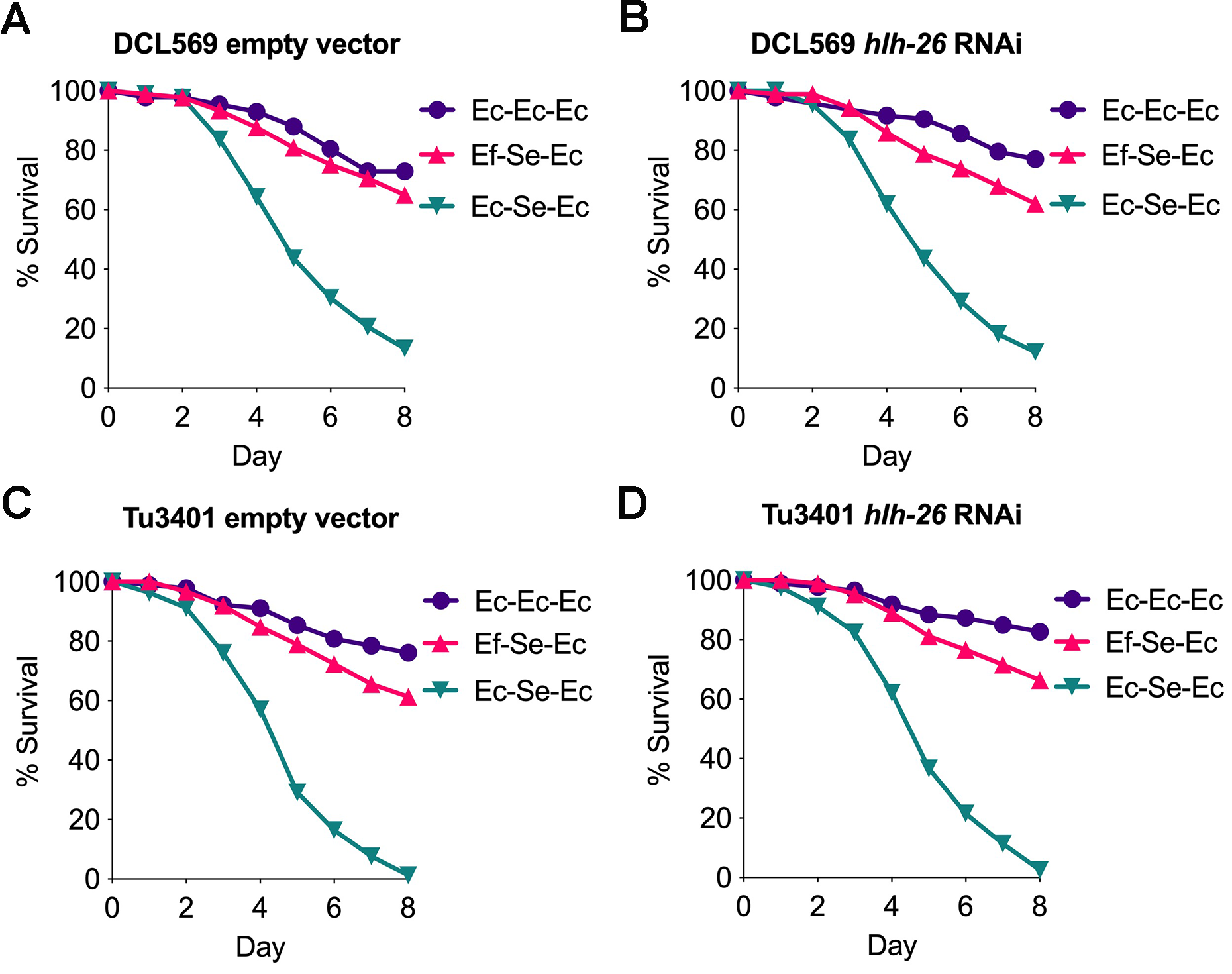

Supplement: S7 Fig — Survival curves assaying E. faecium–mediated protection in the germline-specific RNAi strain DCL569 fed on HT115 expressing (A) empty vector or (B) hlh-26 RNAi animals. Survival curves assaying E. faecium–mediated protection in the neuron-specific RNAi strain Tu3401 fed on HT115 expressing (A) empty vector or (B) hlh-26 RNAi animals. The data underlying all the graphs shown in the figure can be found in S1 Data. RNAi, RNAi interference. (TIF) [file pbio.3001581.s007.tif]

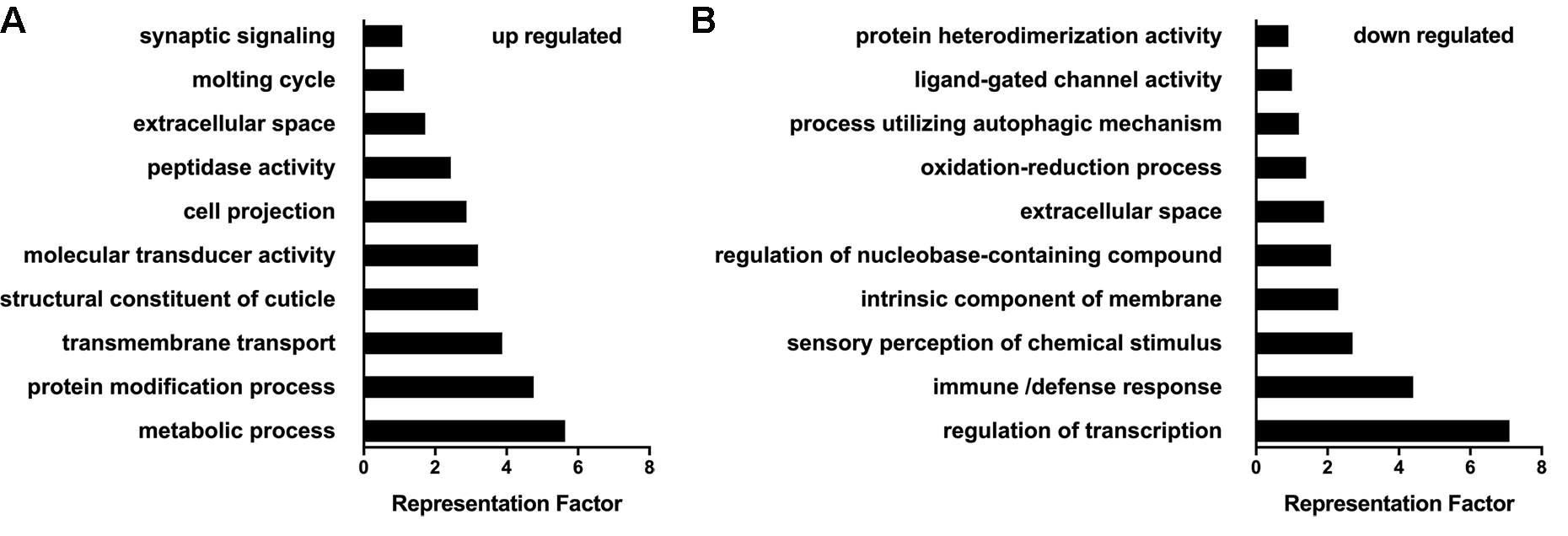

Supplement: S8 Fig — (A) Enrichment analysis of up-regulated genes. (B) Enrichment analysis of down-regulated genes. The data underlying all the graphs shown in the figure can be found in S1 Data. (TIF) [file pbio.3001581.s008.tif]

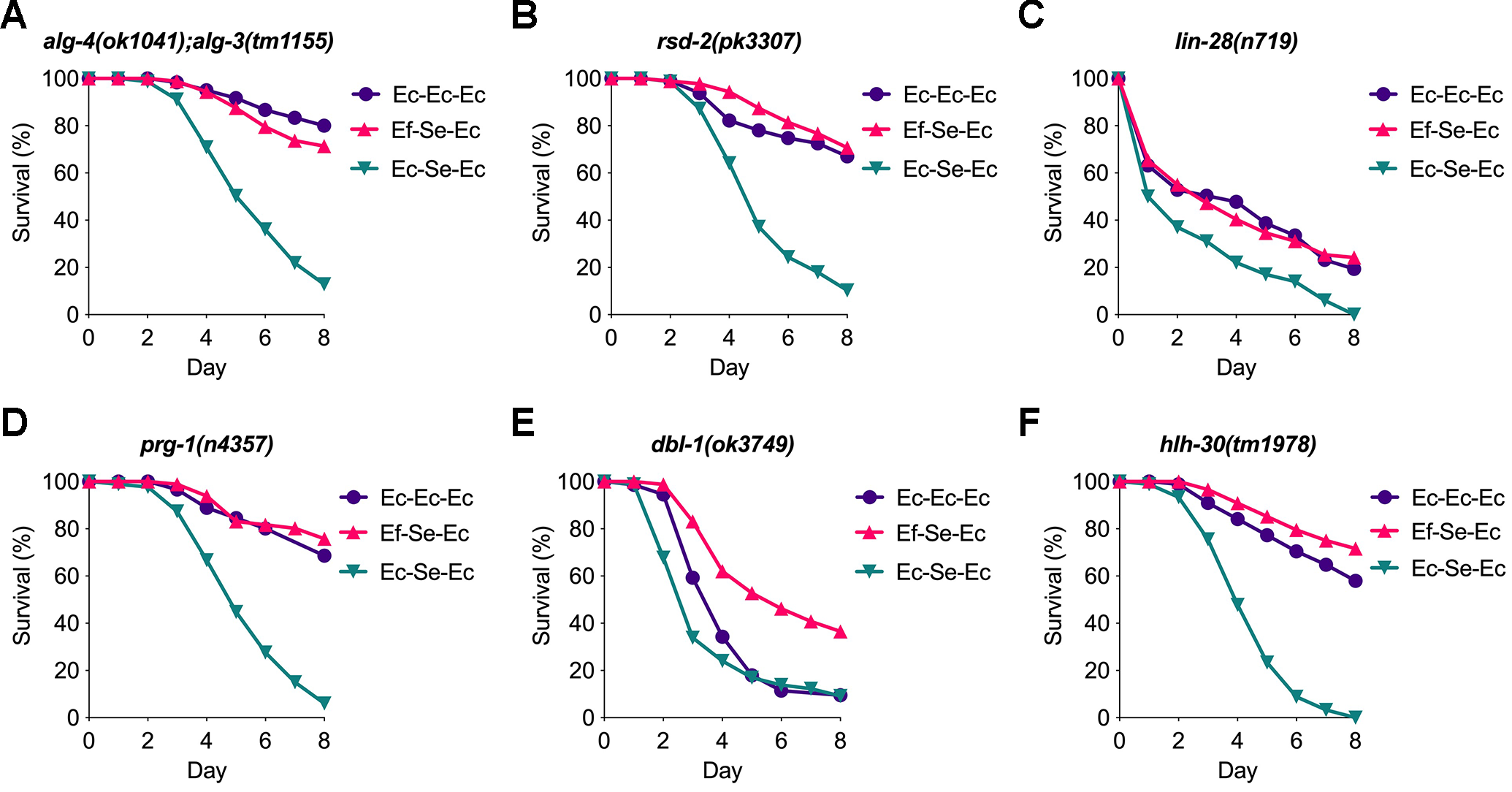

Supplement: S9 Fig — Survival curve assaying E. faecium–mediated protection in (A) alg-4(ok1041); alg-3(tm1155), (B) rsd-2(pk3307), (C) lin-28(n719), (D) prg-1(n4357), (E) dbl-1(ok3749), and (F) hlh-30(tm1978). Survival curves are representative assays of 3 independent experiments. n = 60 to 90. The data underlying all the graphs shown in the figure can be found in S1 Data. Ec, E. coli OP50; Ef, E. faecium; Se, S. enterica. (TIF) [file pbio.3001581.s009.tif]

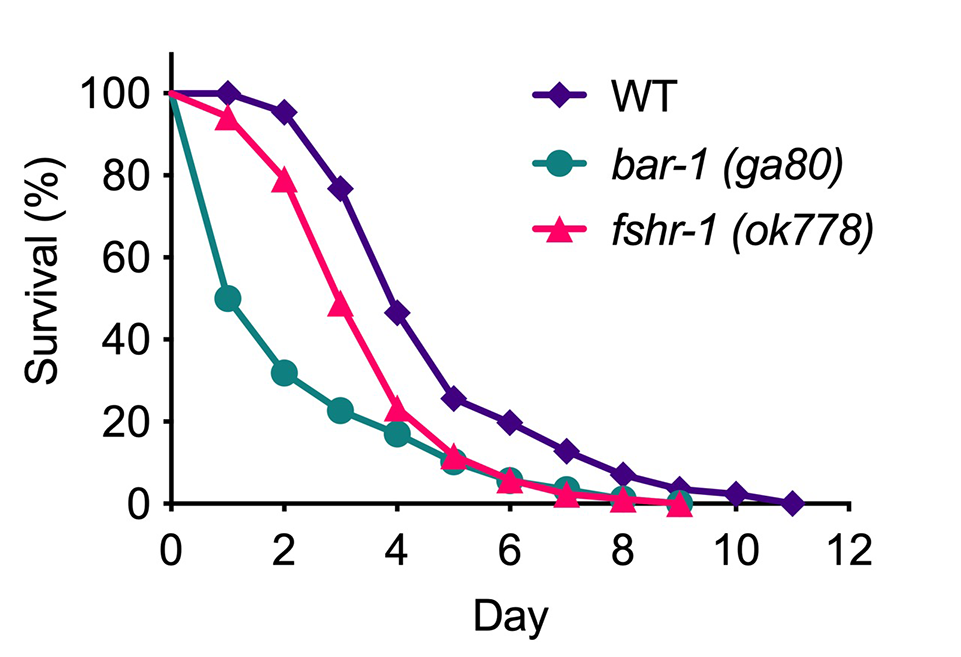

Supplement: S10 Fig — WT, bar-1(ga80), and fshr-1(ok778) animals were grown on modified NGM agar (0.35% peptone instead of 0.25% peptone) for infection with S. enterica and scored for survival. WT animals versus bar-1(ga80), P < 0.0001; fshr-1(ok778), P < 0.0001. fshr-1(ok778) animals versus bar-1(ga80), P < 0.0001. The data underlying all the graphs shown in the figure can be found in S1 Data. NGM, nematode growth medium; WT, wild-type. (TIF) [file pbio.3001581.s010.tif]

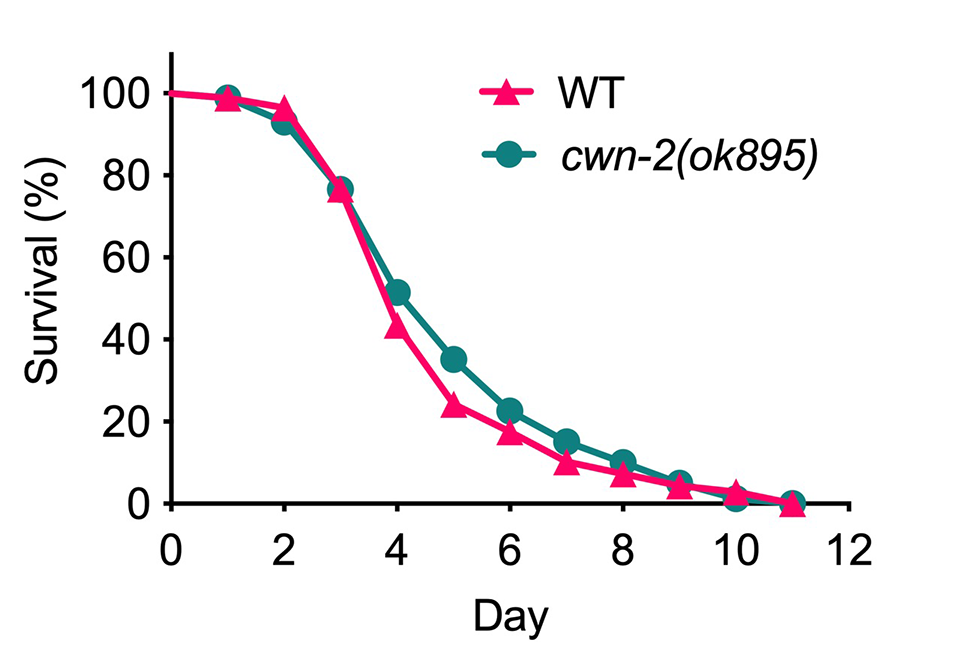

Supplement: S11 Fig — WT and cwn-2(ok895) animals were grown on S. enterica and scored for survival. WT animals versus cwn-2(ok895), P = NS. The data underlying all the graphs shown in the figure can be found in S1 Data. NS, nonsignificant; WT, wild-type. (TIF) [file pbio.3001581.s011.tif]

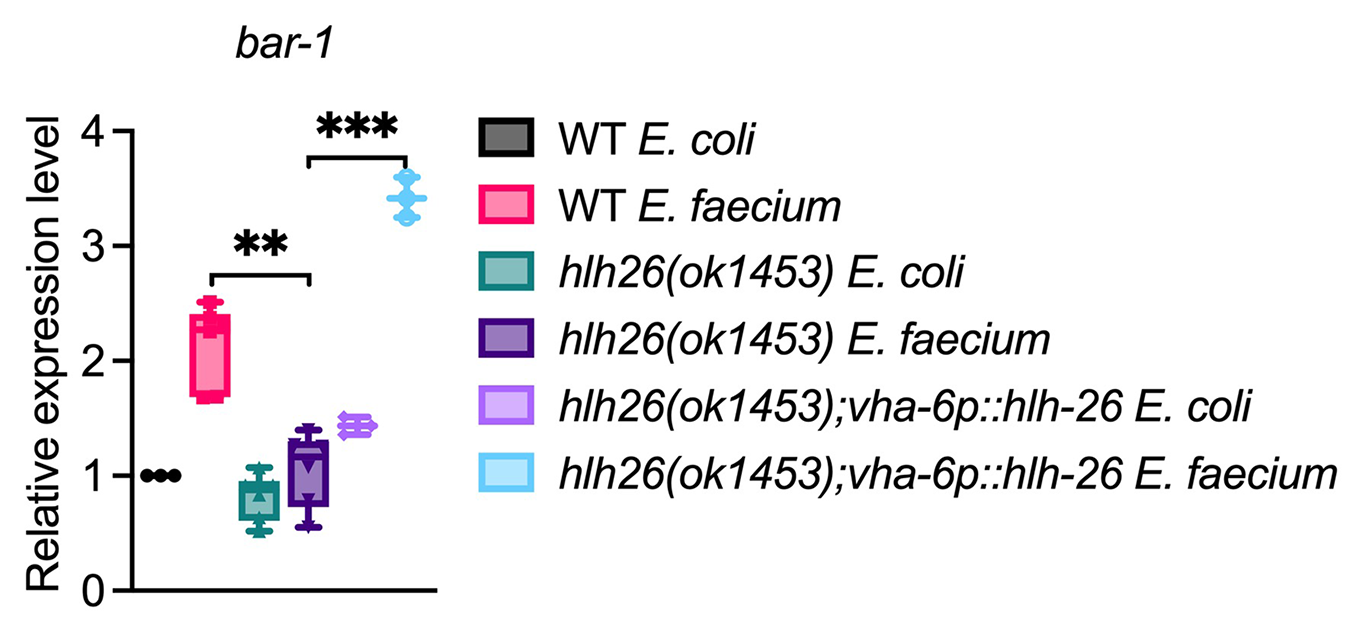

Supplement: S12 Fig — mRNA levels of bar-1 in wild-type, hlh-26(ok1453) animals, and hlh-26(ok1453) animals overexpressing HLH-26 fed on E. coli or exposed to E. faecium were detected using qRT-PCR. Values are expressed as the fold difference compared with wild-type animals fed on E. coli ± SD by one-way ANOVA with Tukey’s multiple comparisons test, **P < 0.01, ***P < 0.001. The data underlying all the graphs shown in the figure can be found in S1 Data. qRT-PCR, quantitative reverse transcription PCR; WT, wild-type. (TIF) [file pbio.3001581.s012.tif]
