# Supplementary material for: The transcription factor HLH-26 controls probiotic-mediated protection against intestinal infection through up-regulation of the Wnt/BAR-1 pathway
Source: PLoS Biol. 2022 Mar 9;20(3):e3001581. doi: 10.1371/journal.pbio.3001581 (PMC8936500; doi:10.1371/journal.pbio.3001581)
Supplement: Attachment — Submitted filename: Response.pdf [file pbio.3001581.s024.pdf]

Response to reviewers

We want to thank the reviewers for their interest in our studies and their thorough review and suggestions. We have responded by performing several additional experiments and by making appropriate changes to the paper. The new studies we have added to the manuscript to address the reviewers' comments are shown in New Table S1 Data, updated Tables S1, S3, S4, S6, S9, and S10, and New Figures 2F, 2G, 2H, 3A, 5C, 5D, 6C, S1, S2, S4, and S12. Changes to the manuscript have been highlighted in gray. We are grateful for the critiques, which have made the paper much stronger, and we hope the reviewers find our responses satisfactory.

Reviewer #1

COMMENT:

Summary

In this study, the authors demonstrated that pretreatment with the probiotic species E. faecium protects C. elegans against S. enterica infection. Through a meta-analysis on the gene expression dataset and transcription factors (TFs), the authors identified 28 TFs that are potentially contribute to such protection. The authors then tested the top 5 candidate TFs and found that the E. faecium mediated protection is dependent on the REF-1 family transcription factor HLH-26. The authors next characterized that HLH-26 is expressed in the intestine and the E. faecium mediated protection is dependent on the intestinal expression of HLH-26. To further describe the HLH-26-dependent E. faecium mediated protection, the authors conducted an RNAseq analysis and identified 3261 differentiated expressed genes by comparing the transcription profiles between hlh-26 mutant worms and wt worms grown on E. faecium. The authors found that many hlh-26-regulated genes were also controlled by many other pathways. To identify which pathways regulates the E. faecium mediated protection, the authors tested the key factors (mutant) in the candidate pathways on their functions in E. faecium mediated protection, and found only bar-1(β-catenin in Wnt signaling) is critical in the E. faecium mediated protection. Next, the authors confirmed that the expression of not only bar-1 but also other Wnt signaling components (cwn-2 and mlg-1), as well as downstream effectors, are all hlh-26 dependent. Finally, the authors validated that the function of bar-1 in E. faecium mediated protection is also hlh-26 dependent. Together, the authors propose a function for the uncharacterized REF-1-family transcription factor HLH-26 in probiotic E. faecium mediated protection against pathogenic S. enterica infection in C. elegans, and that Wnt signaling is a critical component of the HLH-26 immunity program.

I think this manuscript would be suitable for publication PLOS Biology after addressing the several concerns listed below:

RESPONSE: We thank the reviewer for a very good summary of our work.

Major points

COMMENT: *1. It is unclear how the initial analysis was done to get the final number of 28 candidate TFs.*

RESPONSE: We have rewritten the result section to make this clearer (Lines 69-80).

COMMENT: *(a) The identification of the initial 399 genes needs to be described. In S1 table, the 399 genes are from a previous publication (Yuen and Ausubel, 2018), but this literature was not cited in the text, only cited within the supplemental table 1.(a)*
RESPONSE: We have added a description and the corresponding citation in the text (Lines 69-70).

COMMENT: *(b) Within these 399 genes, 244 genes were upregulated, and 155 genes were downregulated (Yuen and Ausubel 2018). It is unclear whether the authors did the co-expression category analysis differently on the up or down regulated genes. It is also unclear if the 30 (out of 125) top ranked co-expressed groups the authors picked to analyze include both up and down regulated genes or just up or down regulated genes. Moreover, the authors may need to specify what algorithm/criteria was used to rank the 125 groups and why the criteria were chosen.*
RESPONSE: We have made it clearer that the 399 genes are up-regulated and down-regulated and that the co-expression category analysis was done using the entire set of 399 misregulated genes. We also clarified that the 30 groups correspond to both up-regulated and down-regulated genes. We selected the top 30 groups because they contain 210 of the 266 genes that are co-expressed (Lines 69-80).

COMMENT: *(c) For the motif analysis, it is unclear how many of the 158 motifs are for each of the 30 co-expressed groups, and how many of the motifs are shared between different groups.*
RESPONSE: We included the corresponding information in S2 and S3 Tables. Each of the 30 co-expressed groups has one or several motifs. For example, group 1 has 5 motifs while group 3 has 9 motifs (S2 Table). 13 motifs are shared between different groups (S3 Table). For example, motif RACACGTGY is shared among groups 2, 3, and 4 (S3 Table).

COMMENT: *(d) Finally, it is unclear how many (and which) of the original 399 differentially expressed genes are (or predicted to be) regulated by the 28 TFs, especially the 5 top TFs tested, and how many of them are up or down regulated by these TFs.*
RESPONSE: We have added the requested information in S3 Table and in the revised manuscript (Lines 80-84).

COMMENT: *(e) Include full list of genes upregulated and downregulated upon exposure to E. faecium in WT and hlh-26 mutant C. elegans with their respective p values. This means that 4 tables need to be added to Suppl information.*
As you present these data, also discuss the immunity genes that are downregulated in C. elegans exposed to E. faecium.
RESPONSE: Because only single samples were used, we do not have p values to include in S4 Table. Ten immune genes were validated by QRT-PCR and the corresponding p values were included (Fig 4B). We have discussed immune genes controlled by HLH-26. The immune genes up-regulated and down-regulated in animals exposed to *E. faecium* are presented and discussed in the previous publication mentioned in our manuscript (Yuen et al. Virulence. 2018;9:683-99. Epub 2018/02/14).

COMMENT: *2. According to the figure caption, Fig 1 B-G are one representative dataset of 3 independent experiments, please provide the survival data of the other 2 independent experiments as a supplemental file, include the statistical analysis of these survival assays (hazard ratio, median survival rate, p-values between conditions, etc.) and how many animals in each survival test. Please provide the results of these for all survival tests throughout the manuscript as well. A detailed experimental procedures and analysis of the survival assays is also missing in the material and method section, the author should provide it since the majority of the data is based on this assay. I would also suggest the author to provide the raw data of these experiments in a separated file.*
RESPONSE: We have provided the requested survival data in S1 Data. We also added detailed experimental procedures and analysis of the survival assays in Material and Methods (Lines 442-452 and Lines 571-574).

COMMENT: *3. Fig 2 A-C seems relatively not as important to be shown as main figure panels, because it simply addresses that the heat killed E. coli has no protection to Se infection, just like live E. coli in Fig 1. I would suggest moving it to a supplemental figure. However, on the other hand, It is very interesting to show if heat killed Efm protect against Se infection or not, and if the protection is from the Efm secreted metabolites or Efm intracellular materials.*
To move Fig 2 A-C to Supp
RESPONSE: We have moved previous Figs. 2A-2C to supplementary figures (current S3 Fig.). We performed the requested experiment and found that heat-killed *E. faecium* also confers protection against *S. enterica* infection, albeit not as strongly as live *E. faecium* (New S1 Fig). (Lines 87-90).

COMMENT: *Also, explicitly describe why in the transcriptomics analyses the authors use heat-killed E coli instead of heat killed E faecium as control. Later discuss heat-stable versus active metabolism as the E. faecium mediators of the probiotic response in C elegans.*
RESPONSE: We analyzed the data from other authors that used heat-killed *E. coli* as a control (Yuen et al. Virulence. 2018;9:683-99. Epub 2018/02/14). As explained in the revised manuscript, we decided to use live *E. faecium* because others have used this approach (Rangan et al. Science. 2016;353:1434-7. Epub 2016/10/07. doi: 10.1126/science.aaf3552. PubMed PMID: 27708039) and because it confers better protection than heat-killed *E. faecium* (New S1 Fig.), indicating that live probiotic is required for full protection. This is discussed in the revised manuscript (Lines 87-90).

COMMENT: *4. It would be interesting to know how many of the 3261 differentially regulated genes share conserved DNA binding motif with HLH-26 to see how many of the genes are likely directly regulated by HLH-26, and what these genes are, and if they are up or down regulated. This information would help to demonstrate if the HLH-26 act as an activator or a repressor, and whether upregulation of immunity is a truly enriched component of the response.*
RESPONSE: The information was added. 543 of 3261 differentially regulated genes share conserved DNA binding motifs with HLH-26 (S4 Table). 183 from the 543 genes are down-regulated in *hlh-26(ok1453)* animals, while 360 are up-regulated in *hlh-26(ok1453)* animals

(Line 216-220). Regarding HLH-26 regulation of immune genes, Fig 4A shows that immune genes are down-regulated in *hlh-26(ok1453)* mutant compared with wild type.

COMMENT: *Also, it would be good to know how many of these 3261 genes overlap with the previous 399 genes, and how many of them overlap with the 70 genes that are predicted to be regulated by HLH-26. This analysis would narrow down the number of the genes and focus the study on HLH-26 directly regulated genes. Furthermore, the author should check the GO cluster of the overlapping genes and the predicted direct HLH-26 regulated genes (specially to check if the immune cluster also show up as highly enriched cluster).*
RESPONSE: We only found that 34 genes overlap with the 399 genes. However, as the reviewer noticed, the study that identified 399 genes used killed *E. coli* as a control while we used live *E. faecium.* Of the 31 downregulated immune genes only 4 (*ilys-2*, *spp-2*, *cnc-2*, *and clec-165*) are predicted to be direct targets of HLH-26 (S6 Table), suggesting that the control of immune genes by HLH-26 is primarily indirect (lines 235-237).

COMMENT: *For the differentially expressed genes that are predicted to be directly regulated by HLH-26 and overlap with the previous 70 genes, the authors may want to verify how many of them are HLH-26 dependent by qRT-PCR on the 4 conditions and to test if they functionally contribute to Efm protection against Se infection through RNAi followed by survival tests (if the genes are up-regulated genes). The results of these tests are likely to provide an actual mechanism of probiotic-mediated immunity against S. enterica, the lack of which is currently my major concern.*
RESPONSE: We disagree regarding the lack of mechanism as we believe our work indicates that HLH-26 controls the Wnt/BAR-1 pathway that is required for probiotic-mediated protection against *S. enterica* infection. Nevertheless, we tested the expression of ten genes that correspond to the subset of 70 HLH-26-dependent genes and found that 7 are HLH-26 dependent (S6 Fig) (Lines 168-170).

We believe that the study of individual HLH-26-direct target genes is outside the scope of this work. Moreover, there is very little evidence in the field of single immune effectors showing phenotypes when mutated or inhibited by RNAi. In this work we decided to focus on HLH-26-dependent genes and found that those that are part of the Wnt/BAR-1 pathway are required for probiotic-mediated protection against *S. enterica*.

COMMENT: *5. Because the analysis on "hlh-26-regulated" genes are not narrowed down to the hlh-26 directly regulated genes as suggested in the last comment, it is not surprise that several other pathways also seem to control the "hlh-26-regulated" genes, and it is not surprise that most of those pathways show no role in the Efm protection (S5 Fig). I would suggest the authors analyze the validated hlh-26 dependent and "directly" regulated genes that contribute to the Efm protection (by RNAi as suggested in last comment), and to check the enriched pathways and the regulation of those genes.*
RESPONSE: As explained in the response above, we believe that the study of direct HLH-26 targets is outside the scope of this work, which focusses on the Wnt/BAR-1 pathway that, as we have established in our work, is HLH-26-dependent.

COMMENT: *6. The authors reasoned the survival of bar-1 animals decreasing on E. coli lawns as*

"susceptible to bacterial infections in general". The authors need to rephrase this since the reduction in survival could be due to Wnt/BAR-1 being required for C. elegans healthspan in general, and not specifically to susceptibility to bacterial infection.

RESPONSE: We rephrased the sentence: "Because *bar-1*(*ga80*) animals were susceptible to bacterial pathogens and even to live *E. coli* (Fig 5A and 5B), we reasoned that animals with a compromised healthspan or susceptible to bacterial infections in general might also be incapable of mounting a protective response against *S. enterica* by pretreatment with *E. faecium.*" (Lines 290-293)

COMMENT: *7. How is E. faecium treatment performed in combination with RNAi? The authors controlled for heat-killed E. coli not acting as a probiotic; however, does live E. coli boost S. enterica resistance? Please test and present these data.*

RESPONSE: We have better explained the conditions used in the revised Method section (Lines 509-510)

Live *E. coli* does not boost *S. enterica* resistance. (Fig 1B – 1G, green lines).

COMMENT: *8. In Fig 5 C-E, the authors try to make comparisons between bar-1 and fshr-1 to address the point that even the bar-1 and fshr-1 both reduce the survival to the same extent without Se infection, bar-1 contribute to the Efm protection against Se infection. The authors would want to include formal statistical analyses to support this claim (instead of a graphical representation).*

RESPONSE: Taking into account comment #9 (below), we replaced previous Figs. 5C-5E (16h *S. enterica* infection rather than 24h). New Figure 5C and 5D shows the survival of *bar-1(ga80)* animals and *fshr-1(ok778)* animals performed using 24h *S. enterica* infection. We have added the survival data of the 3 independent experiments and the statistical data analysis in S1 Data.

COMMENT: *9. Why do the authors modify the conditions to compare bar-1 to fshr-1-deficient worms? Please perform and present fshr-1 experiments in the same conditions than Fig. 1A and 1E.*

RESPONSE: Initially, we were trying different conditions to optimize the assay. We have removed the studies using 16h *S. enterica* infection and only presented the results using 24h *S. enterica* infection.

COMMENT: *Is BAR-1::GFP a rescue construct? Is BAR-1 overexpression sufficient to activate the immune genes upregulated in Fig. 4B? Is BAR-1 predicted to bind to the promoters of those genes?*

RESPONSE: BAR-1::GFP is a rescue construct.

The immune genes listed in Fig. 4B are not predicted to have BAR-1 binding sites nor are they up-regulated by BAR-1 overexpression (Jackson et al. G3 (Bethesda). 2014;4(4):733-47).

COMMENT: *10. In Fig 3A-C, the authors implicate the intestinal function in HLH-26 mediated Efm protection. It is valid to test if the intestinal specific RNAi (MGH171 line) against bar-1 would reduce the survival of Efm treated worms. This condition may also reduce the general detrimental effects of bar-1 since the KD of bar-1 is restricted to intestine.*

RESPONSE: Thank you for the suggestion. *bar-1* RNAi in the intestine significantly decreased the *E. faecium*-mediated protection against *S. enterica* infection (New Fig. 5E and 5F) (Line 298-301).

COMMENT: *11. Present higher-resolution images of hlh-26P::GFP. Current Fig. 3A is confusing because it shows punctate GFP signal, instead of soluble (and hence homogeneous) cytoplasmic expression. As the authors are aware, soluble GFP generated from a promoter fusion is not expected to be trafficked to organelles.*
RESPONSE: We use higher-resolution images. The punctate green signal is from *coel*::RFP used as the co-injection marker. We also showed the *coel*::RFP images in the new figure. (Fig. 3A)

COMMENT: *12. Figure 3D shows rescue but not enhancement of pathogen resistance in animals overexpressing HLH-26. Discuss which would be the bottleneck to enhanced pathogen resistance in this context.*
RESPONSE: We are studying the role of HLH-26 in probiotic-mediated protection and show that its expression rescues the lack of probiotic-mediated protection of *hlh-26* mutant. Our results show that *hlh-26* is not required for pathogen resistance (S5 Fig.). Thus, it is not surprising that its overexpression does not enhance pathogen susceptibility.

COMMENT: *Also, is HLH-26 overexpression in the intestine sufficient to promote bar-1 expression (perform qRT-PCR of bar-1 in this line)*
RESPONSE: We performed the requested experiment. HLH-26 overexpression in the intestine is sufficient to promote *bar-1* expression (New S12 Fig) (Line 359-360).

COMMENT: *13. Could rollers ingest less S. enterica? Please perform survival experiment presented in Fig 1A but comparing WT N2 worms to N2 animals carrying pRF4 rol-6(su1006) alone.*
RESPONSE: The reviewer raised an interesting point, but as explained in the revised Method section, we used non-roller animals in the assays (Line 450-452).

COMMENT: *14. The authors may need to present images of vha-6::HLH-26::GFP worms (without SL2) to define whether HLH-26 is enriched in the intestinal nuclei of C. elegans exposed to E. faecium alone, S. enterica alone, or E. faecium followed by S. enterica exposure.*
RESPONSE: We performed the requested experiment and found that *E. faecium* exposure increases the HLH-26 protein level and nuclear accumulation (New Fig 2G and 2H). We also found HLH-26 nuclear accumulation triggered by *E. faecium* secreted protease SagA (New S4 Fig).

COMMENT: *15. Describe which is the N (independent biological replicates) in Figures 4C & D. Additionally, please check all figure legends to make sure N/n are described in a consistent manner. For example, n=number of animals and N=number of biological replicates.*
RESPONSE: We have made corrections in all figure legends according to n=number of animals and N=number of biological replicates.

COMMENT: *16. The following expression may be inaccurate: "Downregulated genes in hlh-26(ok1453) animals included immune response genes". The data presented in Table S6 show that the analyzed genes were less expressed in hlh-26 than in WT worms, but we do not know if they were further downregulated or not as induced in the hlh-26 KO.*

RESPONSE: The sentence is not present in the revised manuscript.

COMMENT: *In addition to the fold change, the authors may want to present the raw data for all 4 conditions (wild-type animals exposed to E. coli, wild-type animals exposed to E. faecium, hlh-26 animals exposed to E. coli, and hlh-26 animals exposed to E. faecium) for all genes listed in Table S6.*

RESPONSE: We presented raw data for 2 conditions in the S4 Table because we performed RNAseq with only 2 conditions (N2 animals on *E. faecium* and *hlh-26*(*ok1453*) animals on *E. faecium*). We used published expression profiles of animals exposed to *E. coli* and *E. faecium* (Yuen *et al*. Virulence. 2018, 9:683-699). We used the four conditions mentioned by the reviewer in the qRT-PCR experiments.

COMMENT: *Similarly, "...that overlapped with genes that were downregulated in hlh-26 animals (S8 Table)." The genes referred to here may not actually be downregulated; instead, they may be less upregulated. Raw data are needed all around for all datasets to avoid these confusions.*

RESPONSE: We have better explained that the genes are downregulated in *hlh-26* animals compared to wild-type animals exposed to *E. faecium.* S8 Table shows the enrichment analysis. The raw data is shown in S4 Table.

COMMENT: *17. In Fig 6A, the authors show that the activation of bar-1, cwn-2 and mig-1 in Efm is hlh-26 dependent. It would be informative to show if these 3 genes are in the list of up-regulated genes in the RNAseq, and in addition, to show if they share the binding motifs of HLH-26.*

RESPONSE: These 3 genes are not in the list of down-regulated genes in the RNAseq because they are down regulated less than 2-fold. The RNAseq study shows several Wnt target genes down-regulated more than 2-fold in *hlh-26(ok1453)* animals compared to N2 animals (Table S4 / Downregulated Wnt targets). None of the three genes contain HLH-26 binding motifs.

COMMENT: *18. In Fig 6C, the authors show the increasing BAR-1::GFP signal through Efm treatment in a hlh-26 dependent manner. It would be informative if the authors can show zoom in images with higher resolution to show the nuclear localization of BAR-1, since the activation of Wnt canonical signaling should induce the nuclear localization of BAR-1/β-catenin.*

RESPONSE: We repeated the experiment using higher magnification. Overall, BAR-1::GFP levels increased by *E. faecium* treatment in an *hlh-26* dependent manner and BAR-1::GFP accumulated in the nuclei of animals exposed to *E. faecium* compared to animals exposed to *E. coli*. (New Fig 6C).

COMMENT: *The author may also show the cellular localization of the POP-1/TCF as the transcription factor regulated by Wnt signaling.*

RESPONSE: POP-1/TCF cellular localization was not shown because expression level of *pop-1* did not change. The RNAseq results show that the expression level of *pop-1* was not different in *hlh-26* animals than wild-type N2 animals (0.929 fold, Table S4). We also confirmed with qRT-PCR that expression level of *pop-1* did not change in *hlh-26* animals compared with wild-type N2 animals (Fig 6A).

COMMENT: *19. The authors state: "We provided evidence indicating that HLH-26-dependent immune genes were activated upon exposure to E. faecium to defend against S. enterica infection". However, by the end of this manuscript it is still unclear which is the mechanism by which HLH-26 mediates E. faecium enhanced resistance against S. enterica.*
Which of the defense effector genes upregulated in Fig. 4B contribute to E. faecium-enhanced resistance to S. enterica but not to resistance to single S. enterica infection? Meaning, which of those or other genes mediate probiotic-enhanced immunity in C. elegans?
So far, the authors present correlations between expression of genes downstream of HLH-26 and enhanced immunity; however, the only molecular players demonstrated to mediate the enhanced immunity are HLH-26 and BAR-1, and BAR-1 is a known mediator of the immune response in C. elegans and other organisms. Further, X et al already showed that bar-1-deficient C. elegans are compromised in their ability to mount a response to E. faecium exposure.
If the authors would shift the focus of their manuscript to presenting a function for a yet uncharacterized C. elegans transcription factor, then they may want to introduce the notion that HLH-26 seems to be a nematode-specific transcription factor. Hence, considering that the introduction focuses on the impact of probiotics on human health, the authors may want to elaborate on which lessons HLH-26 teach us that may be translatable to other organisms.
RESPONSE: We believe that the focus of the manuscript is appropriate. Very few immune effector genes acting downstream transcription factors and main regulators of immune response play a role in defense. Thus, we did not focus our study on genes downstream HLH-26. Our manuscript presents the first evidence that both HLH-26 and BAR-1 are required for probiotic-mediated protection. We believe our work provides a novel mechanistic understanding of the probiotic protection against gut infections and establishes a new REF-1/Wnt axis that is involved in antimicrobial defense in the gut. HLH-26 is a REF-1 family member of basic helix-loop-helix transcription factors that are widely found in metazoans. Finally, we provide the first demonstration that a REF-1 family member of basic helix-loop-helix transcription factor controls the conserved Wnt pathway.

Minor points
COMMENT: *1. Please add the line and page numbers to the manuscript and address the points by referring to the line and page numbers.*
RESPONSE: We have added line and page numbers.

COMMENT: *2. The author should specify the E. coli strains used in each experiment (e.g. OP50, HT115).*
RESPONSE: We have added the strains in the Figure legends.

COMMENT: *3. Fig 4B showed the qPCR data using bar graph. Box plot would be the better choice. Please*

go through all other figures/supplemental figures as well to change the bar graph to box plots.
RESPONSE: We have changed all qPCR data with box plot. (Fig 4B, 6A, 6B and S6, S12 Fig)

COMMENT: *4. In material and method, under statistical analysis, "ns" indicates non-significant. However, in all the Figure legends, non-significant is written as "NS". Check to make sure it is consistent.*
RESPONSE: We have made the corrections.

COMMENT: *5. Part of the conclusion of section 1 "and that HLH-26 was required for E. faecium-activated protection against S. enterica infection." Is demonstrated by experiments shown earlier in the section but not by the experiments showing that 7/10 e faecium-induced genes are hlh-26 dependent. Thus, decouple the conclusion from the QRT results and make it a general section conclusion or move this half of the conclusion upwards.*
RESPONSE: We made the suggested change (Line 138-139).

COMMENT: *6. Because you are not looking at the protein, and much less its activity, using a promoter fusion, replace "...foci of HLH-26 activity" for "tissues in which hlh-26 is expressed"*
RESPONSE: Thanks. Replaced. (Line 180)

COMMENT: *7. In a similar note, the following is an overstatement: "The intestinal function of HLH-26 in protection against S. enterica infection was confirmed using strain MGH171, which allows intestine-specific RNAi." Because, although unlikely for a transcription factor, formally a an mRNA can be produced in the intestine, which will be abrogated by intestinal RNAi, whereas the protein may be exported and exert its critical function in a different tissue. In te absence of a protein function he authors need to continue using expressions as "suggest" or "is in line" instead of confirms.*
RESPONSE: We have changed “confirmed” to “suggested”. (Line 182).

COMMENT: *8. Word missing here" Because BAR-1 plays a critical role in the response to ? and…"*
RESPONSE: We have made corrections. Now it reads: “Because BAR-1 plays a critical role in the response to *E. feacium* and …” (Line 405)

COMMENT: *9. Acknowledgment has a typo*
RESPONSE: We have made corrections.

COMMENT: *10. The authors state: "The primer sequences are available upon request and are presented in S10*
RESPONSE: We have made corrections. (Line 550)

COMMENT: *11. Table.". Please list all primers in table S10.*
RESPONSE: Added the missing primers.

COMMENT: *12. The authors state: "The animals were then visualized for bacterial load using a Leica M165 FC fluorescence stereomicroscope" However, no bacterial load experiments are presented here.*

RESPONSE: A correction was made: “The animals were then visualized using a Leica…” (Line 555)

Review #2

COMMENT: *In this study, Sang and colleagues set out to identify mechanisms underlying E. faecium-mediated protection against Salmonella enterica infection using the model C. elegans. Through analyses of co-expressed genes during E. faecium exposure and predicted common binding motifs, the authors identified a requirement for hlh-26 in E. faecium-mediated protection against S. enterica infection. The authors further characterized the role of HLH-26 in this process through the use of transcriptional reporters, tissue-specific knockdown/rescue, mRNAseq, and gene enrichment analyses. They nicely showed that HLH-26 functions in the intestine to control this response and functions with the Wnt/BAR-1 pathway in this context. This manuscript logically follows the authors' previous publications on the regulation of intestinal immunity in C. elegans, and presents conclusions that are supported by the data presented.*

Overall, I believe that the authors' have provided sufficient evidence that HLH-26 and the Wnt/BAR-1 pathway are involved in E. faecium-mediated protection in this S. enterica infection model. I have a few questions for the authors consideration:

RESPONSE: We thank the reviewer for a very good summary of our work and for highlighting the importance of the findings. We have addressed the specific critiques below.

COMMENT: *1. Is HLH-26 activated in response to the proteolytic activity of SagA, a secreted protease from E. faecium, which was previously shown to control the protection of C. elegans against S. enterica (PMID: 27708039)? I think experiments to address this question should be included both to add mechanistic insight to the story, and also to reconcile the proposed mechanisms with what is already known about E. faecium-mediated protection against bacterial infection.*

RESPONSE: We performed the suggested study and found that HLH-26 is activated in animals exposed to *E. coli* BL21 expressing SagA. We also found that the SagA-mediated protection is HLH-26-dependent (New S4 Fig) (Lines 132-139)

COMMENT: *2. I am confused about the rationale for the experiments comparing the protection between heat-killed E. coli and live E. faecium. Is examining killed E. faecium on the protection against S. enterica a better experiment? In other words, do heat-killed E. faecium also provide resistance against S. enterica?*

RESPONSE: We compared protection between heat-killed *E. coli* and live *E. faecium* because we started our work using a dataset that used those conditions (Yuen *et al*. Virulence. 2018, 9(1):683-699). We performed the requested experiment (role of killed *E. faecium* on the protection against *S. enterica*) (New S1 Fig.). Heat-killed *E. faecium* provides resistance against *S. enterica* albeit the protection is not as strong as that conferred by live *E. faecium* (New S1 Fig). Both live *E. coli* and heat-killed *E. coli* do not provide resistance against *S. enterica* (Fig 1B and S1 Fig) (lines 87-90).

COMMENT: *3. The authors demonstrate that hlh-26(ok1453) have reduced protection in the Efm-Se-Ec*

infection model. However, Figs. 2C, 2F, and 2H illustrate that the protection is not fully hlh-26-dependent and other factors may be important in E. faecium protection. Do ref-1;hlh-26 double mutants have complete abrogation of protection?

RESPONSE: We performed the requested experiment and found that the *ref-1/hlh-26* double mutant does not show a stronger protection effect than the single *hlh-26(ok1453)* mutant (New S2 Fig) (Lines 95-97).

COMMENT: *4. The title for Figure 2 does not properly represent the data shown.*
RESPONSE: We have updated Fig 2 as well as the title. The new title reads "*hlh-26* is required for probiotic *E. faecium* mediated protection against *S. enterica* infection".

COMMENT: *5. Although the authors conclude that select innate immune genes are dependent on hlh-26 (Fig. 4B), the relevance of these innate immune genes in the protection against S. enterica is unclear. The authors could additionally show the difference in S. enterica CFUs between wild-type N2 and hlh-26(ok1453) after prophylaxis with E. faecium to demonstrate that these innate immune genes may be involved in the eradication of S. enterica. This experiment may not yield clean results, but perhaps is worth trying.*
RESPONSE: Viable *S. enterica* colony-forming units (CFUs) recovered from lysed worms revealed a ~10 folds increase of *S. enterica* colonization in *hlh-26(ok1453)* animals compared to N2 animals after one day of infection (Fig 2F) (lines 127-131).
