# Supplementary material for: The transcription factor HLH-26 controls probiotic-mediated protection against intestinal infection through up-regulation of the Wnt/BAR-1 pathway
Source: PLoS Biol. 2022 Mar 9;20(3):e3001581. doi: 10.1371/journal.pbio.3001581 (PMC8936500; doi:10.1371/journal.pbio.3001581)
Supplement: Attachment — Submitted filename: Response to reviewers.pdf [file pbio.3001581.s025.pdf]

Response to reviewers

We would like to again thank the reviewers for their interest in our work and reviewer 1 for the additional critiques. New changes to the manuscript have been highlighted in gray.

Reviewer #1

COMMENT: *The authors have satisfactorily addressed most of my concerns. Before publication, it would be necessary to address the remaining concerns listed below:*

RESPONSE: We have addressed the specific critiques below.

COMMENT: *1) Explicitly state in the main text that the RNAseq data comes from a SINGLE replicate; therefore, several false negatives and false positives are expected.*

RESPONSE: We mentioned that single samples were used in the main text (lines 249-250): "*Because individual samples were used for RNAseq, which can provide false candidate genes, we verified by qRT-PCR the hlh-26-dependent regulation…*"

COMMENT: *2) Need to revise conclusions for hlh-26-dependent induction of some of the tested genes. For example, contrary to the conclusion, C04G6.5 in Fig. 4 is induced in the hlh-26 mutant to a similar extent as in the WT worms. The difference is that the max level induction is lower because hlh-26 affects the basal expression of this gene; however, the delta of expression E. faecium to E. coli is still large in the absence of hlh-26. Therefore, it cannot be said that hlh-26 is required for C04G6.5 induction. What the data says is that HLH-26 is required for C04G6.5 basal expression and may subtly contribute to C04G6.5 induction. The same is the case for other genes including cwn-2 & R02E4.1 (Fig. 6) and C54D10.3 (Suppl. Fig. 6).*

RESPONSE: We modified the manuscript to address this issue (lines 254-257, and line 313-314): "*Because HLH-26 also affected the basal expression of C04G6.5 and cnc-11 (Fig 4B), it is unclear to what extent the reduced expression of these two genes in hlh-26(ok1453) animals exposed to E. faecium is due to the role of HLH-26 their expression under control conditions or in the presence of E. faecium.*" "*HLH-26 is also required for basal expression of cwn-2 (Fig 6A).*"

COMMENT: *3) In response to the previous comment "Is BAR-1 overexpression sufficient to activate the immune genes upregulated in Fig. 4B? ", the authors responded: "… The immune genes listed in Fig. 4B are not predicted to have BAR-1 binding sites nor are they up-regulated by BAR-1 overexpression (Jackson et al. G3 (Bethesda). 2014;4(4):733-47)."*
However, the fact that BAR-1 is not sufficient to activate the immune genes is not discussed in the manuscript. As a central player in the proposed model, BAR-1's insufficiency needs to be discussed.

RESPONSE: We believe that we have sufficiently discussed Wnt/BAR-1 in the context of the experiments we performed (lines 384-386 and 410-430). While interesting, we believe further discussion should be part of a review/preview article.

COMMENT: *Similarly, the previous comment on POP-1 was not fully addressed. POP-1 nuclear localization is controlled via its phosphorylation status. Therefore, even if pop-1 is not transcriptionally regulated, the localization of the POP-1 protein would be informative of its ON-OFF status. The authors need*

to at least discuss this point.
RESPONSE:
We further discussed this issue in the revised manuscript (lines 314-316): "*The expression level of pop-1, which encodes a Wnt effector that together with BAR-1 regulates gene expression, was not affected by E. faecium exposure or the absence of hlh-26 (Fig 6A). However, we cannot rule out an effect on the posttranslational activation of POP-1.*"

COMMENT: *4) To reach a broader readership, replace N2 with WT in text and all figures*
RESPONSE: We replaced the text and figures.

COMMENT: *5) Describe exactly the number of base pairs cloned upstream of the ATG to generate the promoter fusion constructs and the primer sequences used. Do the same for the protein fusions; provide coordinates and primers used to make the constructs.*
RESPONSE: We added the requested information (Lines 482-483, 489-491, 499-501).

COMMENT: *6) In Supplementary tables depicting GFP quantification, authors need to indicate the number of animals used to calculate the median/mean shown in the tables and disclose whether the depicted values and mean or medians. As of now, there is a single value per biological replicate.*
RESPONSE: We included the number of animals, mean value, and SD (S1 Data/Tables 2H, 4D and 6D).

COMMENT: *7) Include the TF/s that bind to each gene in Table S3 worksheets named "28 TF controlled genes" & "5YF controlled genes".*
RESPONSE: We added the information to Table S3.

Typos or similar
COMMENT: *1. Across the manuscript, please change all qPCR instances for qRTPCR (because what is being measured is mRNAs)*
RESPONSE: Replaced all qPCR instances by qRT-PCR.

COMMENT: *2. Legend Fig.3A: the construct is referred to as HLH-26::GFP, but the image shows hlh-26P::GFP.*
RESPONSE: Corrections were made in the figure legend.

COMMENT: *3. Title Fig 4: HLH-26 loss of function regulates an innate immune transcriptional program.*
RESPONSE: The new title reads: HLH-26 regulates an innate immune transcriptional program.

COMMENT: *4. Lines 250-252: : "We tested verified by qRT-PCR the hlh-26-dependent regulation of ten genes involved in immune response (S6 Table) under four conditions: wild-type animals exposed to E. coli, wild type animals exposed to E. faecium, hlh-26(ok1453) animals exposed to E. coli, and hlh-26(ok1453) animals exposed to E. faecium.*
RESPONSE: We removed "tested".

COMMENT: *5. Legend Fig. 5: Delete the Euro sign in "..expressing € empty.."*
RESPONSE: Deleted it.
